# Mechanism Study of Proteins under Membrane Environment

**DOI:** 10.3390/membranes12070694

**Published:** 2022-07-07

**Authors:** Yue Zhang, Xiaohong Zhu, Honghui Zhang, Junfang Yan, Peiyi Xu, Peng Wu, Song Wu, Chen Bai

**Affiliations:** 1 School of Life and Health Sciences, School of Medicine, The Chinese University of Hong Kong, Shenzhen 518172, China; zhangyue@cuhk.edu.cn (Y.Z.); zhuxiaohong@cuhk.edu.cn (X.Z.); 221059040@link.cuhk.edu.cn (H.Z.); 220059035@link.cuhk.edu.cn (J.Y.); 117030076@link.cuhk.edu.cn (P.X.); 2 School of Chemistry and Materials Science, University of Science and Technology of China, Hefei 230026, China; 3 Warshel Institute for Computational Biology, Shenzhen 518172, China; 4 School of Biomedical Engineering, Health Science Center, Shenzhen University, Shenzhen 518055, China; 2150242005@email.szu.edu.cn; 5 South China Hospital, Health Science Center, Shenzhen University, Shenzhen 518116, China; 6 Chenzhu Biotechnology Co., Ltd., Hangzhou 310005, China

**Keywords:** membrane protein, conformational changes, coarse-grained model

## Abstract

Membrane proteins play crucial roles in various physiological processes, including molecule transport across membranes, cell communication, and signal transduction. Approximately 60% of known drug targets are membrane proteins. There is a significant need to deeply understand the working mechanism of membrane proteins in detail, which is a challenging work due to the lack of available membrane structures and their large spatial scale. Membrane proteins carry out vital physiological functions through conformational changes. In the current study, we utilized a coarse-grained (CG) model to investigate three representative membrane protein systems: the TMEM16A channel, the family C GPCRs mGlu2 receptor, and the P4-ATPase phospholipid transporter. We constructed the reaction pathway of conformational changes between the two-end structures. Energy profiles and energy barriers were calculated. These data could provide reasonable explanations for TMEM16A activation, the mGlu2 receptor activation process, and P4-ATPase phospholipid transport. Although they all belong to the members of membrane proteins, they behave differently in terms of energy. Our work investigated the working mechanism of membrane proteins and could give novel insights into other membrane protein systems of interest.

## 1. Introduction

Membrane proteins, encoded by approximately 25% of the human genome, are essential for various biological processes [1], such as signal transduction, molecule/ion transport, immune recognition, electrical signal modulation, and catalysis [2,3,4,5]. Membrane proteins are classified as ion channels, receptors, and transporters [6]. Nearly ~60% of drugs [7,8] target membrane proteins. To design novel effective drugs and reveal the molecular mechanisms underlying drug activity, it is essential to deeply dig into the working mechanism of membrane proteins at a molecular level. Mainly, a membrane protein undergoes dynamic conformational changes between several discrete conformation states to carry out its function actively [9]. Therefore, structural details of the membrane protein are always focused on during its working process.

To investigate the mechanism of membrane proteins, experimental researchers usually try to obtain membrane protein structures at an atomic resolution. Due to a low natural abundance and toxicity when overexpressed [10,11], resolving the stable functional membrane protein structures is challenging. With the advancements in X-ray, NMR, and cryo-EM [12], it is possible to measure membrane protein structures with high resolution. Nevertheless, since only static structural information is obtained by experiments, elaborating the molecular mechanism of membrane proteins during their dynamic working process is challenging. Computational simulations have remarkable advantages for exploring the working mechanism of membrane proteins, which could build the membrane protein model at a molecular level and predict the effects of structural dynamic perturbation on membrane protein function. However, most computational approaches face difficulty simulating large-scale membrane protein systems with thousands of residues and membrane molecules. Additionally, capturing the dynamic information during the working process of membrane proteins, especially energy barriers and transition state structures, is difficult.

Considering the large size of membrane protein systems and dynamic features during the working process, in the current work, we constructed three representative membrane protein systems (TMEM16A chloride channel, family C GPCRs mGlu2 receptor, and P4-ATPase phospholipid transporter) using a coarse-grained (CG) model developed by Arieh Warshel [13,14,15]. We investigated their workflow under the membrane environment. For most protein systems, the electrostatic effect contributes to various kinds of interactions [16,17]. Hence, an accurate description of the electrostatic term is of great significance in drawing energy profiles for proteins. The electrostatic-based CG model is an effective and accurate method for large-scale biophysical systems [18,19,20] and is widely used in many membrane protein systems such as β2AR-Gs [19], SARS-CoV2-spike [21], Hv1 proton channel [22], etc. Additionally, the developed CG model would give dynamic features and energy profiles for the conformational changes of membrane proteins. Our results revealed the dynamic information for the three membrane protein systems during the working process based on energy and provided unique insights in the molecular mechanism for other membrane protein systems.

## 2. Methods

### 2.1. Model Assembling

Modeller [23,24] was utilized to construct major discrete structures for three membrane protein systems. The PDB structures include: 5OYG and 7B5C for the TMEM16A channel system, 7EPA, 7EPB, and 7E9G for the mGlu2 receptor system, and 6K7G, 6K7J, 6K7K, 6K7N, 6K7L, and 6K7M for the P4-ATPase transporter system. Intermediate structures that connect them were generated by targeted molecular dynamics (TMD). For these structures, membrane particles were added, and the solvent was treated implicitly. Extensive MD relaxation using Molaris-XG software 9.15 (created by Arieh Warshel, USC, Los Angeles, CA, USA) [25,26] was carried out until the convergence was achieved.

### 2.2. Coarse-Grained (CG) Model, Monte Carlo Proton Transfer (MCPT) Algorithm, and the Calculation Process of Folding Free Energy

The coarse-grained (CG) model developed by Arieh Warshel gives a reliable description of protein stability and functions and considers the importance of the electrostatic effects of proteins [25]. In the CG model, the side chain is reduced to a simplified united atom and each residue’s backbone atoms are treated explicitly. The total energy of the CG is:

(1) $$\Delta G_{fold}^{CG}=\Delta G_{side}^{CG}+\Delta G_{main}^{CG}+\Delta G_{main-side}^{CG}\;$$

here, $\Delta G_{side}^{CG}$ and $\Delta G_{main}^{CG}$ represent the main chain and side chain contributions, respectively, and $\Delta G_{main-side}^{CG}$ represents a total protein and side-chain flexibility in estimating the overall conformational entropy.

The main chain energy involves backbone solvation ($\Delta G_{solv}^{CG}$) and the hydrogen bonds interaction ($\Delta G_{HB}^{TOTAL}$):

(2) $$\Delta G_{main}^{CG}=c_{2}\Delta G_{solv}^{elec}+c_{3}\Delta G_{HB}^{TOTAL}\;\;$$

Scaling coefficients *c_2_* and *c_3_* are 0.25 and 0.15, respectively, whereas the main chain/side chain coupling term consists of the electrostatic ($\Delta G_{main-side}^{elec}$) and the van der Waals parts ($\Delta G_{main-side}^{vdw}$):

(3) $$\Delta G_{main-side}^{CG}=\Delta G_{main-side}^{elec}+\Delta G_{main-side}^{vdw}\;$$

The side chain term is expressed by electrostatic, polar, and hydrophobic interactions, and the van der Waals component.

(4) $$\Delta G_{side}^{CG}=\Delta G_{side}^{elec}+\Delta G_{side}^{polar}+\Delta G_{side}^{hyd}+c_{1}\Delta G_{side}^{vdw}\;$$

So, the total energy equals to the sum of all these terms, among which the side chain term is essential since it is involved in many energy interactions.

(5) $$\;\Delta G_{fold}^{CG}=c_{1}\Delta G_{side}^{vdw}+c_{2}\Delta G_{solv}^{CG}+c_{3}\Delta G_{HB}^{CG}+\Delta G_{side}^{elec}+\Delta G_{side}^{polar}+\Delta G_{side}^{hyd}+\Delta G_{main-side}^{elec}+\Delta G_{main-side}^{vdw}\;$$

It should be noted that, the treatment of electrostatic effects in this CG model is a key factor in clearly explaining the energetics in many complicated and large biological systems [19,27,28]. It is computed as a sum of the change in free energy associated with charge–charge interactions between ionizable side chains. The electrostatic contribution of the side-chain atoms is:

(6) $$\Delta G_{side}^{elec}=-2.3RT\underset{i}{\sum }Q_{i}^{MC}*\left(pKa_{i}^{i}-pKa_{i}^{w}\right)+\Delta G_{Q}^{dev}+\Delta G_{QQ}^{f}-\Delta G_{QQ}^{uf}\;$$

$pKa_{i}^{i}$ and $pKa_{i}^{w}$ are the pKa of the ith ionizable residue in protein and water, individually. $Q_{i}^{MC}$ is the charge of the ***i***th residue in the given ionizable state. Both the $Q_{i}^{MC}$ and $pKa_{i}^{i}$ were obtained using the MC approach. $\Delta G_{Q}^{dev}$ is a correction term, reflecting the scaled-down effect of the change in an ionizable residue protonation state upon unfolding. $\Delta G_{QQ}^{f}$ and $\Delta G_{QQ}^{uf}$ are the free energies of the charge–charge interactions in folding and unfolding proteins, respectively [15].

Monte Carlo proton transfer (MCPT) is a reliable method to estimate the pKa value of ionizable residues in the protein environment [29,30]. The MC procedure involves the proton transfer between ionizable residue pairs or the ionizable residue along with the bulk. During each MC move, the electrostatic free energy of the folded protein, $\Delta G_{elec}$, for the ***m_th_*** charge configuration of the ionizable protein residues is: [31]

(7) $$\Delta G_{elec}^{m}=-2.3RT\underset{i}{\sum }\langle Q_{i}\rangle *\left(pKa_{i}^{i}-pH\right)+\Delta G_{QQ}^{m}$$

where 〈***Q_i_***〉 is MC averaged charge. $pKa_{i}^{i}$ is the pKa value of the ionizable residue. The charge configuration is acceptable if the electrostatic free energy reaches a lower value or satisfies the Metropolis criteria.

For membrane protein modeling, the membrane grid has a regular spacing between membrane particles. To modulate the membrane protein energetics instead of modelling membrane thickness fluctuation or phase behavior, the membrane grid is not modified during CG energy calculations. Membrane particles near the protein atoms are built. The membrane grid is treated with continuous derivatives (Supplementary materials text S1), as discussed in previous studies [14].

When calculating the energy, the membrane is considered, a typical representation is the treatment of self-energy $\Delta G_{self}$, which is one part of electrostatic energy contribution for the sidechain and is related to the charge value of each ionizable group in its surrounding environment.

(8) $$\Delta G_{self}=\sum_{i}(U_{self}^{np}\left(N_{i}^{np}\right)+U_{self}^{p}\left(N_{i}^{p}\right)+U_{self}^{mem}\left(N_{i}^{mem}\right))$$

In the formula, ***U*** means the effective potential, ***i*** runs over all ionizable residues, $U_{self}^{np},\;U_{self}^{p}$, and $U_{self}^{mem}$ are energy contributions from non-polar residues, polar residues and membranes atoms, respectively, $N_{i}^{np},\;N_{i}^{p},\;\mathrm{and}\;N_{i}^{mem}$ refer to the number of non-polar residues, polar residues, and membrane atoms in the surrounding of the ith ionizable residue.

**Figure 1:**
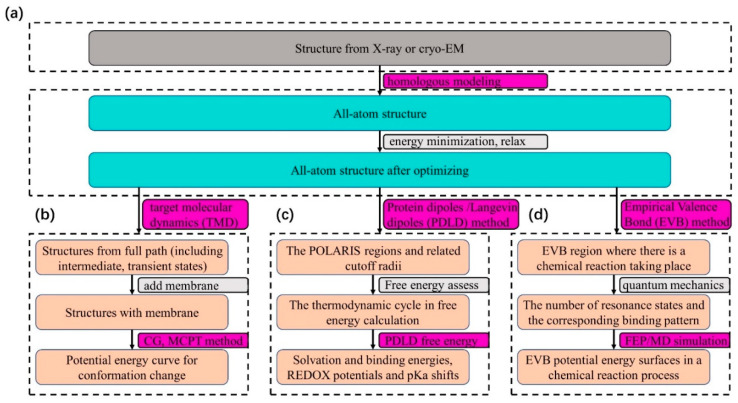
The general way of using Molaris to get free energy in different biological processes. (**a**) The procedures to optimize the structures before TMD, PDLD, and EVB calculation. The path to obtain (**b**) the folding free energy of conformational changes; (**c**) the solvation and binding energies, REDOX potentials, and pKa shifts by PDLD method; (**d**) the activation energy along with potential energy surfaces reflecting chemical reaction process by EVB method.

As shown in Figure 1, the calculation process of folding free energy consists of the following steps: model construction, acquisition of intermediate conformations, determination of charge configuration, and conformation free energy calculation.

We assembled major protein complex states by obtaining available experimental structures from the Protein Data Bank database followed by homology modeling by Modeller [23,24] and optimizing the structures with energy minimization and “relax” (Figure 1a). Then, TMD simulations were used to generate intermediate conformations of the optimized structures. We added the membrane particles for each intermediate structure. Finally, each conformation was converted into a CG model that was then used to calculate the folding free energy (Figure 1b and Figure S1). For each intermediate structure, the electrostatic charge of ionizable residues is determined by the MCPT method, and the energy is calculated using the Equation (5).

### 2.3. Protein Dipoles/Langevin Dipoles (PDLD) Method and PDLD Energy Calculation Process

The PDLD model represents the protein by an all-atom model with the solvent around the protein by a grid of Langevin-type dipoles. The model divides the protein into four regions: Region I contains the group of molecules of interest (e.g., the substrate), Region II contains the rest of the explicit molecular system (e.g., the protein), and Region III contains the solvent (the Langevin dipoles) in and around Region I and Region II. The bulk solvent around Region III (Region IV) is represented by a dielectric continuum. To obtain stable results, Region III is divided into inner and outer grids where the spacing of the inner grid is usually smaller than the average spacing between the solvent molecules. The default radius value for the inner shell is 15 Å and the radius for the outer shell should cover all atoms in Region I and Region II. The protein dipoles/Langevin dipoles (PDLD) method and its variants can be used to study solvation and binding energies, REDOX potentials, and pKa shifts. For example, a specified thermodynamic cycle is defined to calculate the free energy of a particular biological process to obtain the ionization energy of a charged group or the binding energy of a ligand [32].

As shown in Figure 1c, to obtain PDLD free energy, we should define four POLARIS regions representing different parts of interest and a thermodynamic cycle for the given biological process after structure optimization. Finally, we could obtain more information such as binding energy and pKa shifts.

### 2.4. Empirical Valence Bond (EVB) Method and EVB Energy Calculation Process

The EVB method is a simple and effective quantum mechanical/molecular mechanical method. A postulated mechanism (for instance, proton transfer, nucleophilic attack) can be translated into a force field that the computer can understand and can be used for calculating the free energy profile [33]. In the EVB method, the classical force field is used to simulate the parts of the protein removed from the actual chemical reaction since there is no bond breaking or making in this region. In the chemical reaction region of the protein, a quantum mechanical empirical method is used to represent the changes in the reaction atoms’ electronic (as opposed to nuclear) coordinates. After a few steps, the activation energy (**∆*G***) can be obtained by running a series of trajectories on potential surfaces, which gradually drive from one valence bond state into another [34].

The procedures to obtain the potential energy surface and activation energy ***(*∆*G***) are depicted in Figure 1d. After structure optimization, the quantum atoms that change their bonding pattern as the reaction process and the type of the atoms in each resonance form and their charge in each resonance form should be defined first. Next, we define the bonding pattern in each of the resonance forms. After this, the program will automatically compute the parameters of the EVB force field and we can obtain potential energy surface of the EVB system and the activation energy (**∆*G***). All simulations are performed by the Molaris-XG package 9.15 (created by Arieh Warshel, USC, Los Angeles, CA, USA) [25,26].

## 3. The Gating Mechanism of TMEM16A Ion Channel

Ion channels are transmembrane glycoprotein pores that modulate ion conduction across the cell membrane. These channels consist of distinct subunits encoded by an individual gene. According to the ways of activation, most ion channels are subdivided into two classes: voltage-gated and ligand-gated channels. Voltage-gated ion channels are activated or inactivated by changing membrane potentials. Ligand-gated ion channels are precisely modulated by specific ligands. The binding of the ligand to a separate site of the ion channel induces conformational changes of the binding site, which propagates to the channel pore to open the channel gate and allow ion conduction. Thus, the study of conformational changes has great significance for understanding the working mechanisms of ion channels. Free energy profiles of conformational changes can give a detailed dynamic explanation during the activation process of ion channels. In this work, TMEM16A, a ligand-gated chloride channel activated by the binding of two Ca^2+^ [35,36,37] was selected as an example to investigate its activation mechanism.

**Figure 2:**
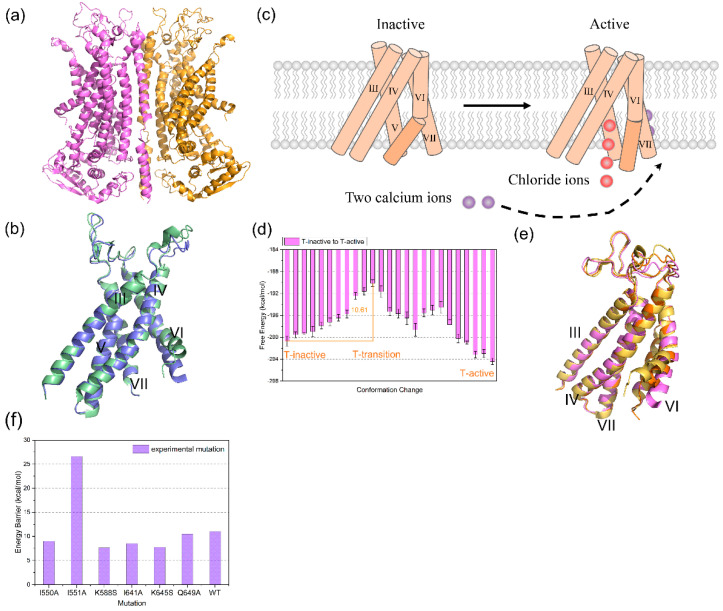
The activation process of TMEM16A and the free energy profile of the process. (**a**) The homodimer TMEM16A structure (PDB: 5OYG). Two subunits are, respectively, colored violet and orange. (**b**) Chloride channel of the TMEM16A subunit with an all-atom model. The inactive channel is colored pale green (PDB: 5OYG) and the active channel is colored slate (PDB: 7B5C). (**c**) Schematic description of the TMEM16A chloride channel activation and Cl^-^ conduction across the membrane. (**d**) The description of free energy profiles in the conformational changes of TMEM16A activation process in the absence of calcium. The energy barrier was presented as orange lines and the error bar colored black. T-inactive, T-transition, and T-active represents the inactive, transition, and the active state of TMEM16A, respectively. (**e**) Protein structure of TMEM16A channel. Three major states are indicated: inactive state (yellow), transition state (orange), and active state (purple). (**f**) Mutational effects on energy barrier of TMEM16A activation process. The barrier was calculated by energy barrier = G (transition) − G (inactive).

As shown in Figure 2, TMEM16A is a homodimer membrane protein with nearly 2000 residues. Each subunit contains a chloride channel formed by helix III–VII. The activation of each TMEM16A channel is dependent on two Ca^2+^ bindings. In the absence of Ca^2+^, the electrostatic repulsion between the vacant Ca^2+^ binding site and helix VI leads to the loosening of helix VI far from the Ca^2+^ binding site and towards helix IV, favoring the access of Ca^2+^ [38]. The binding of Ca^2+^ starts the activation process and changes the negatively charged environment, inducing large-scale conformational changes, including helix VI tightening and opening of the channel pore to allow Cl^−^ ion conduction across the membrane (Figure 2c).

Representation of free energy profiles is a great way to help deeply understand the activation process of the TMEM16A channel. Due to the high cost of computational resources, it is challenging work to describe the energy profiles. In this work, we utilized Molaris-XG software 9.15 (created by Arieh Warshel, USC, Los Angeles, CA, USA) [25,26] to construct CG models of TMEM16A channels to reduce the computational cost. The initial structure models of two conformations of TMEM16A were constructed by the Cryo-EM structures (PDB: 5OYG, representing the inactive state; PDB: 7B5C, representing the active state) solved by Paulino et al. [38,39]. Subsequently, a series of intermediate conformations between the inactive state and active state by TMD were constructed. Then, we described the free energy profiles formed by these conformations (Figure 2d). The energetic profiles could explain the behaviors of TMEM16A activation process.

Referring to the energy profiles, the free energies of inactive, transition, and active states are −200.74 kcal/mol, −189.72 kcal/mol, and −203.95 kcal/mol, respectively. T-active has the lowest free energy. The free energy of T-inactive is more than T-active. It indicates that the active state of TMEM16A is more stable. The T-active state has great physiological significance in the subsequent Cl^−^ ions conduction, which is closely related to electrical signals and transport pathways [39,40]. During the activation process, the free energies of the conformations increase until reaching the peak, representing the transition state. Then, the free energies decline generally (Figure 2d). The free energy barrier of the conformational changes is 10.61 kcal/mol (Figure 2d). As shown in Figure 2e, compared with the three states, the overall conformations are similar, except for the conformational difference at the intracellular half of helix VI. From inactive to transition to active state, the intracellular half of helix VI generally straightens and keeps away from helix IV. Then, these structural changes open the channel.

We calculated mutational effects on the energy barrier of conformational changes of TMEM16A (Figure 2f). Our results revealed that the free energy barriers of the activation process for I550A, K588S, I641A, and K645S were smaller than that in the wild system, suggesting these mutations favor TMEM16A activation. The energy barrier of I551A is larger than the wild system, meaning that I551A impedes the channel’s activation. For Q649A, the barrier is approximately equal to that in the wild system. Our results are supported by Dutzler’s lab [40,41]. They estimated single-channel current (i) and open probability (Po) from non-stationary noise analysis and proposed that I550A and I641A increase in Po, I551A decreases in Po, and Q649A is close to the wild system in Po [41]. I550A and I641A stabilize the open pore state and promote the activation process of the TMEM16A channel, contrary to I551A. Additionally, Dutzler’s lab also explored the activation properties of pore mutants, K588S and K645S by concentration–response relations [40] and found that K588S and K645S enhanced activation potency. This is consistent with our data that the energy barrier of the activation process for K588S and K645S is lower than that in the wild system.

Furthermore, we predicted the reaction energy difference (G_inactive_ − G_active_) in Table S1. The difference in the wild system is 3.21 kcal/mol. For mutations that decrease the energy barrier of activating TMEM16A (I550A, K588S, I641A, and K645S), the reaction energy differences are larger than 3.21 kcal/mol, suggesting that the active states are more stable than the inactive states in the mutants. The energy barrier from the inactive state to the transition state is smaller than that from the active state to the transition state. The pathway from the inactive state to the active state is more accessible than the opposite. For Q649A, the reaction energy difference is only 1.83 kcal/mol, smaller than 3.21 kcal/mol in the wild system, indicating that the stabilization of the inactive state resembles the active state and the energy barrier from the inactive to the transition state is approximate to that from the transition state to the inactive state. In comparison to the wild system, the reaction energy difference of I551A drops obviously. The inactive state is infinitely more stable than the active state. The energy barrier of the pathway from the inactive state to the transition state is higher than the opposite pathway. It gives an additional explanation that for I551A, the energy barrier of the activating channel is larger than that in the wild system. Although Dutzler’s lab provides no reaction energy difference for these mutations, we predicted the associated results and looked forward to experimental support. Our current study is in an early stage. In future, we will further investigate the effects of more residue mutations and validate their significance through experiments.

## 4. The Transduction Mechanism of mGlu2 Receptor

G protein-coupled receptors (GPCRs) are the largest family of membrane proteins. Since GPCRs are involved in nearly all physiological processes, they are also the key targets for current drug development [42,43]. Among all GPCRs, family C GPCRs are structurally unique, function as constitutive dimers, and have multiple structural domains. Metabotropic glutamate receptors (mGlus) are family C GPCRs that play key roles in the central nervous system. For example, it can slow the neuro modulatory effects of glutamate and tune synaptic transmission and excitability [44].

**Figure 3:**
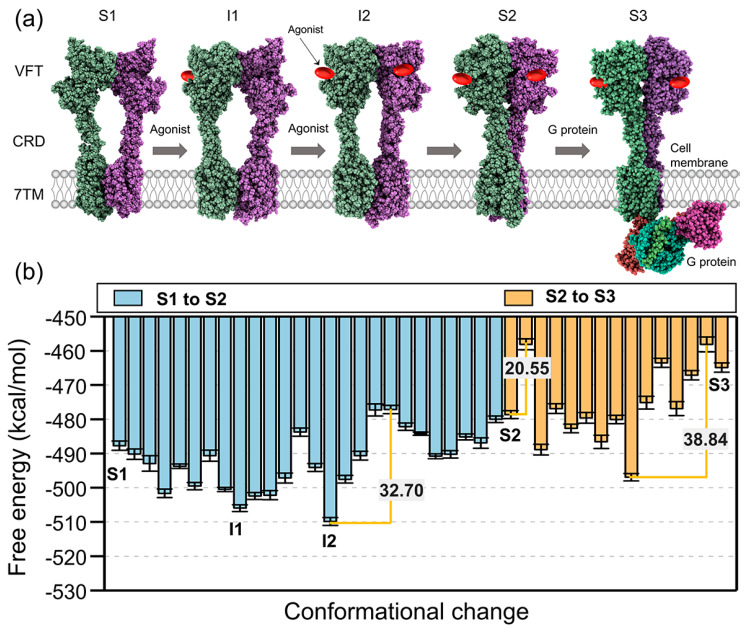
(**a**) The schematic diagram for conformational changes of the mGlu2 activation pathway. The structures of state “S1”, “S2”, and “S3” are models of mGlu2 in the inactivated, agonist-bound, and fully activated states, respectively. These three structures are built by Cryo-EM structures (PDB ID: 7EPA, 7EPB, 7E9G). States “I1” and “I2” are the intermediate structure obtained from our calculation. (**b**) The CG free energy profile for the conversion between the three major states (S1, S2, and S3). The blue bars correspond to the free energy changes between S1 and S2, and the orange bars correspond to the S2 to S3. I1 and I2 correspond to the two local minimizations between S1 and S2. The energy barriers are shown in orange.

The mGlus possess a relatively large extracellular domain (ECD). The ECD incorporates a Venus flytrap (VFT) domain, containing the orthosteric binding site for native ligands, and a cysteine-rich domain (CRD), which connects the VFT domain and a 7-transmembrane (7TM) domain (Figure 3a) [45,46,47]. Furthermore, researchers found that the large-scale conformational changes underlie the transmission of signals from the VFT domain to the 7TM in the membrane using X-ray crystallography and cryo-electron microscopy [48,49,50,51,52,53,54,55]. Glutamate or agonist binding at the VFT domain (sensory domain) closes to the VFT lobes and results in the rearrangement of the dimer interface of the VFT domains from ‘inactivate’ (state S1) to ‘activate’ (state S2). Next, this conformational change is thought to bring the adjacent CRDs closer together to activate the G protein-binding interface (Figure 3a, state S3). However, the energy basis of the activation mechanism of mGlus remains unknown.

The mGlu2 has gained attention as a drug target for schizophrenia and depression treatment [56,57]. Understanding the molecular mechanism of mGlu2 activation is vital for drug discovery. In this work, we constructed CG models of mGlu2 to investigate the conformational changes during the activation process of mGlu2. The structural models of three conformations of mGlu2 homodimers were built by Cryo-EM structures (PDB ID: 7EPA, 7EPB, 7E9G) resolved by Lin et al. [50] and Seven et al. [53]. Next, we generated a series of intermediate structures between these three major states using TMD. Then, we picked structures at equal intervals to reproduce the free energy profiles (see Methods for details). Figure 3b depicts the CG free energy profile of the conformational transition between the three states.

The calculated conformational free energies for the state “S1”, “S2” and “S3” are −487.79 kcal/mol, −478.67 kcal/mol, and −464.89 kcal/mol, respectively. Among these three experimental conformations, state “S1” has the lowest energy, state “S2” is the second highest, and state “S3” is the highest. In the “S1” state, the ECDs of the VFT domains adopt an open conformation, and the CRD and 7TM domain do not interact with each other. The energy barrier between the state “S1” and “S2” is 32.70 kcal/mol (blue bars in Figure 3b). Once the agonists bind to the VFT domains of the mGlu2 homodimers, the VFT domains close, bring the CRDs near, and rotate almost 180° of 7TM in the membrane. From our calculation (Figure 3b), we found that two local minimizations between state “S1” and state “S2”. First, one agonist binds to a single VFT domain, the free energy readily goes down, and forms the first stable intermediate state (state “I1” in Figure 3b, −509.89 kcal/mol). This phenomenon is consistent with the conclusion that during the activation process of mGlu2, a conformation in which one VFT domain is ‘closed’ and the other is “open” is observed [51]. Later, the other VFT domain of mGlu2 binds another agonist, and overcomes the 22.20 kcal/mol energy barrier, forming another stable intermediate structure (state “I2” in Figure 3b). The agonist binds to both VFT domains and forms a stable intermediate, but the 7TM domains remain in an inactivated conformation. To facilitate G-protein coupling, the 7TM undergoes a further reorientation to introduce an asymmetric dimer interface (state “S3”). A comparison of “S2” and “S3” states reveals two important energy barriers, 20.55 kcal/mol, and 38.84 kcal/mol, respectively (orange bars in Figure 3b) between these two states.

## 5. The Transport Cycle of P4-ATPase Flippase

**Figure 4:**
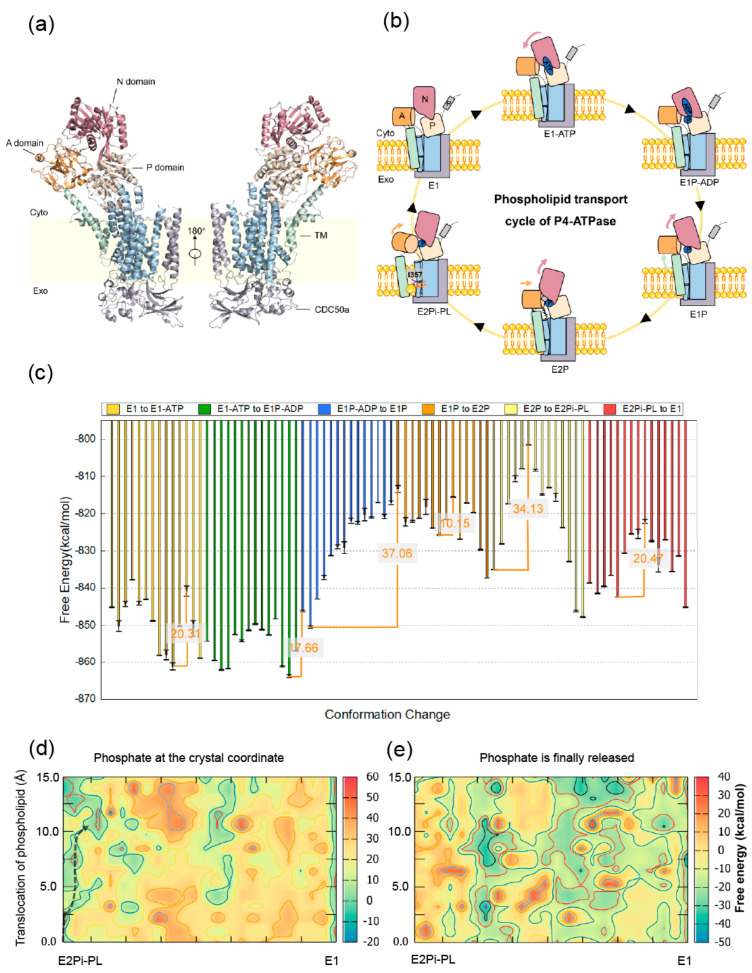
(**a**) Overall structure of P4-ATPase. (**b**) The general mode of the P4 flippase reaction cycle. Cyto represents the cytoplasmic side. Exo represents exoplasmic side. (**c**) The CG free energy profile of the conformational transition between the six states. The energy barriers are shown in orange. (**d**) Coupled free energy map of the conformational change of the system, the phosphate release, and the phospholipid translocation when phosphate is at the crystal coordinate. The barrier along the black route is 23.71 kcal/mol. (**e**) Coupled free energy map of the conformational change of the system, the phosphate release, and the phospholipid translocation when phosphate is finally released.

In living eukaryotic cells, phospholipids are unevenly distributed in the biological membranes [58,59]. The cytoplasmic leaflet has a high concentration of phosphatidylethanolamine (PE) and phosphatidylserine (PS), whereas the extracellular leaflet is rich in phosphatidylcholine (PC) and sphingolipids [60]. The asymmetric distribution of phospholipids involves many important biological processes, such as inflammatory responses, axonal regeneration, and myotube formation [61]. Although phospholipids can move laterally in the bilayer membrane, the translocation of polar head groups of phospholipids through the hydrophobic intrinsic membrane is energetically unfavorable [62]. Type 4 P-type ATPase (P4-ATPase) plays a crucial role in active phospholipid transportation by flipping lipids from the outer leaflet to the inner leaflet [63,64]. P4-ATPase is a heterodimer composed of a catalytic α-subunit, and an ancillary β-subunit [65]. The α-subunit has three cytosolic domains involved in the ATPase catalytic cycle: the actuator (A), nucleotide-binding (N), phosphorylation (P) domains, and ten transmembrane (TM) helices (Figure 4a). The β-subunit, cell cycle control protein 50a (CDC50a), has two transmembrane helices and a large exoplasmic loop, playing an important role in the stability of the transporter complex [66,67]. The general mode of the P4 flippase reaction cycle proposes that the enzyme exists in six main intermediates, E1, E1-ATP, E1P-ADP, E1P, E2P, and E2Pi-PL [68] (Figure 4b). Intracellular ATP binds to the nucleotide-binding site in the apo state E1, leading to the proximity of the N and P domains by acting as a bridge, generating E1-ATP. Then, the phosphorylation of ATP leads to the E1P-ADP state. Subsequently, ADP is released from the N domain, generating the transient phosphorylated state E1P. The approaching of the A and N domains, collaborating with the penetrating C-terminal regulation between the P and N domains, forms the E2P state. Finally, the forcing-out A domain contributes to the phospholipid-binding state E2Pi-PL, followed by a conformational transition to the apo state E1 [68] (Figure 4b). However, little is known regarding the energy profile and the detailed coupling mechanism of the catalytic process by P4-ATPase.

Here, we carried out a systematic study of free energy changes that P4-ATPase undergoes during the lipid translocation process. The initial models were built on the cryoelectronic microscopy structures of P4-ATPase reported by Hiraizumi et al. in six transport states (PDB ID: 6K7G, 6K7J, 6K7K, 6K7N, 6K7L, 6K7M) [68]. Due to the size and complexity of the system, we seeked to investigate the catalytic process of P4 flippase by coarse-grained models developed by Warshel and his colleagues [69]. Then, the CG simulation workflow steps were processed to obtain the microscopic energy landscape of the conformational transition between the six states. The relative free energies of all the six end-point states are −845.16, −854.24, −846.11, −813.39, −835.02, and −838.66 kcal/mol, respectively. What can be seen is that the free energies are relatively lower in E1, E1-ATP, and E1P-ADP states, compared with that in the remanent assemblies E1P, E2P, and E2Pi-PL states. After ADP is released from the E1P-ADP state, the N domain retreats from the association with the P domain and the A domain can rotate, forming the transient phosphorylated E1P state [68]. Particularly high free energy occurs in the E1P state, consistent with the experimental observation that the E1P state is conformationally unstable and of high energy [70,71]. The conformation change in E1P may further trigger the rearrangement of the protein system, which results in the relatively high free energy of the E2P and E2Pi-PL states [67,72]. Our results highlight the importance of the rotation and intrusion movements of the A and N domains after the release of ADP [68].

However, the six stabilized structures do not directly reveal the conformational transition in the reaction process. The intermediate structures might be too unstable to be captured by experimental means. Therefore, we resorted to the target molecular dynamics method to generate the intermediate structures between each pair of endpoint states, such as E1→E1-ATP. The CG free energy profile of the conformational transition between the six states is presented in Figure 4c. The results indicate that the energy fluctuations in the transition processes for E1→E1-ATP and E1-ATP→E1P-ADP are relatively lower than that of other transition processes. During E1P-ADP→E1P, there is a steady increase in free energy for the intermediate structures, with the highest energy barrier of 37.06 kcal/mol. Then, the tendency shows a slight decline and a lower energy barrier (10.15 kcal/mol) occurs for E1P→E2P. The second largest energy barrier (34.13 kcal/mol) happens for E2P→E2Pi-PL, with a sharp rise and then a gradually dropping energy change.

The energy needed to be overcome for E2Pi-PL→E1 is relatively low, with the value at 20.47 kcal/mol. There is no consensus on which step is rate-limiting for P4-ATPase yet. Mateusz et al. found that the E1P state formation is rate-limiting for the transport of the Listeria monocytogenes Ca^2+^-ATPase using the single-molecule FRET method [73]. Our current study indicates that this is a valid possibility for P4-ATPase, because the highest energy barrier happens when E1P-ADP E1P. The relatively high energy barrier for the transition E2P→E2Pi-PL may be explained by the approach of the head group of phospholipids [74].

In the CG simulations described above, extensive energy details were obtained for the unbound protein conformations, without a quantitative description of how the phosphate dissociation, lipid translocation, and conformational change affect each other. To this end, we endeavored to get the free energy map that couples the conformational change, the phosphate release, and the phospholipid transport by the PDLD/s-LRA/β method (Figure 4d,e). There was a significant difference between the two conditions: for the coupled free energy of the conformational change of the system, the phosphate release and the phospholipid translocation when phosphate is at the crystal coordinate, the initial values are low then there is a noticeable increase with the structural change proceeding. The barrier along the route is 23.71 kcal/mol (Figure 4d). While for the coupled free energy, when phosphate is finally released, the free energy was high at the very start of the conformation change, then decreased (Figure 4e). The tendency reflects that it is energy favorable for the crystal phosphate to bind to the initial protein structures during the transition of E2Pi-PL to the E1 state, but it is not prone to bind to the remaining transition structures. This represents that the phosphate may release at the early time when the E2Pi-PL transforms to E1. This can be validated by the energy unfavorably binding with the initial structures when the phosphate is at a relatively far distance (Figure 4e). According to Hiraizumi et al., the dephosphorylation is associated with lipid transmembrane translocation, and the phosphate release may couple with the phospholipid translocation by allowing further stretching of the M1–M2 helices [68]. Our computational results provide a quantitative explanation for those assumptions. Those findings further improve the understanding of P4-ATPase, which can lead to the design and control of lipid translocation processes at the molecular level. For phosphate release and phospholipid transport in P4-ATPase, even with progress in X-ray crystallography and cryo-electron microscopy, atomic level structural information on the intermediate states involved in the overall P4-ATPase functional dynamics is still very limited [63,68,75]. According to our free energy calculation, phosphate tends to dissociate at the beginning during the translocation of the phospholipid, which is consistent with Hiraizumi’s work [68]. More detailed studies of the coupling mechanism of the conformational change, the dissociation of phosphate, and the dislocation of phospholipids will be addressed in subsequent work.

## 6. Conclusions

In the current study, we seek to explore the working process of three representative membrane protein systems utilizing the consistently developed CG model [14,15]. We determined the energy barrier of the working process by free energy analysis and identified the proper reaction pathway according to the amount of energy barrier. The CG model has an advantage in large-scale biomacromolecule systems rather than all-atom models. It should be noted that the electrostatic term contributes most in biomacromolecule systems. A correct description of the electrostatic term has great importance for investigating its mechanism. The CG model utilized in this work emphasizes the electrostatic effects and the solvation of ionizable residues. Our current work captured the conformational changes and determined the energy barrier of the working process by CG profiles. Our results explain the mechanisms of the three membrane protein systems in terms of free energy. Although the three systems belong to membrane proteins, they show different behaviors.

The development of high-speed atomic force microscopy (HS-AFM) makes it possible for experimentalists to visualize channels and transporter transitions in physiological environments at the single molecule level [76]. It provides conformational information to help understand the mechanism of membrane proteins. For example, Marchesi et al. examined the conformational dynamics of cyclic-nucleotide gated (CNG) channels by HS-AFM and observed the cyclic nucleotide-binding domains approach the membrane and splay away from the four-fold channel axis accompanied by a clockwise rotation with respect to the pore domain [77]. Ruan et al. observed membrane reconstituted GltPh at work and found that transport was mediated by large amplitude 1.85-nm “elevator” movements of the transport domains [78]. HS-AFM helps observe more details of conformational changes in protein systems. However, such an observation missed the key information on the transition states and reaction energy barriers. The developed CG model can well address the issue. By combing HS-AFM and the large-scale CG method, the researchers can further understand the mechanism of gating-related structural transition at an atom level, such as CNG channels. They belong to complex modular proteins, modulated by membrane voltage and nucleotides binding [79]. The details of the coupling are still unclear [80]. A multipronged approach, combining HS-AFM, CG, and functional experiments may be helpful in answering this issue. HS-AFM provides conformational details. CG methodology constructs the complete path of the conformational changes and determines the overall energy profiles. These findings could illuminate the coupling mechanism from structural and energetic perspectives. Then, functional measurements can provide powerful validations. We believe these efforts may have the potential to illustrate this long-standing issue and advance the field beyond the current bottlenecks.

In the membrane protein system, the membrane environment places physical constraints on protein and has a profound effect on the efficiency of the working cycles [9]. The protein isolated from the native membrane by experiments could only provide partial information. Important membrane properties that affect the structure and function of membrane systems include electrostatics [81], lateral tension [82], and hydrophobic matching [82]. The membrane environment tightly modulates protein function by altering these properties. Thus, although it is more tractable to investigate the structure and function only based on discrete conformations without the membrane bilayer, it is deficient. In our current work, we fully considered the possible implications of membranes for the working mechanism of membrane proteins and built the complete models including the membrane environment by theoretical and computational modeling. Our work has potential value for illustrating the energetic mechanism of complex membrane protein systems. The method of this work may be also appropriate for other biophysical systems.

## Data Availability

Data sharing is not applicable.

## Supplementary Materials

The following supporting information can be downloaded at: https://www.mdpi.com/article/10.3390/membranes12070694/s1, Figure S1: Representation of the structures sampled from the CG model with membrane environment. The membrane particles are colored grey. Table S1: The free energy of mutations with inactive state and active state (kcal/mol).
