# Supplementary material for: Mechanism Study of Proteins under Membrane Environment
Source: Membranes (Basel). 2022 Jul 7;12(7):694. doi: 10.3390/membranes12070694 (PMC9322369; doi:10.3390/membranes12070694)
Supplement: Supplementary file 1 [file membranes-12-00694-s001.zip › membranes-1790917-supplementary.pdf]

Supplementary Materials

S1. Side chain van der Waals interactions.

ΔG_{side}^{vdw} describes the van der Waals interactions between simplified side chains, composing of the interactions between both protein residue simplified side chains $\Delta G_{side-side}^{vdw}$ and the interactions between side chains and membrane grid atoms $\Delta G_{side-mem}^{vdw}$. $\Delta G_{side-side}^{vdw}$ is given by:

$$\Delta G_{side-side}^{vdw} = \sum_{i<j} \epsilon_{ij}^{0} \left[3\left(\frac{r_{ij}^{0}}{r_{ij}}\right)^{8} - 4\left(\frac{r_{ij}^{0}}{r_{ij}}\right)^{6}\right] \quad \text{(S1)}$$

$$\epsilon_{ij}^{0} = \sqrt{\epsilon_{i}^{0}\epsilon_{j}^{0}} \quad \text{and} \quad r_{ij}^{0} = \sqrt{r_{i}^{0}r_{j}^{0}} \quad \text{(S2)}$$

ϵ_{ij}^{0} and r_{ij}^{0} define the well depth and equilibrium distance, separately. These parameters were refined by minimizing the root-mean-square deviations between the calculated and observed values of both the atomic positions and protein sizes.

The treatment of van der Waals interaction between side chains and membrane grid atoms is different to allow for efficient modeling of the membrane effect. A continuous membrane is built to reduce the need to generate a new grid when the protein is displaced or changes its structure. Because of the fact that membrane grid should be deleted upon direct contact with the side chain atoms, the van der Walls interaction between the protein and the membrane is given by:

$$\Delta G_{side-mem}^{vdw} = \sum_{i<j}\left[\frac{A_{ij}}{\left(\alpha + r_{ij}^{6}\right)^{2}} - \frac{B_{ij}}{\alpha + r_{ij}^{6}}\right] \quad \text{(S3)}$$

Here A_{ij} and B_{ij} are parameters for interacting i^{th} side chain and j^{th} membrane grid atom, r_{ij} represents the distance between side chain atom and membrane grid, and α is a vdW cutoff parameter, equaling to 7452.75 $Å^{6}$.

$$A^{ij} = 4\,\epsilon_{ij}^{0}\,(r_{ij}^{0})^{12} \text{ and } B^{ij} = 4\,\epsilon_{ij}^{0}\,(r_{ij}^{0})^{6} \quad \text{(S4)}$$

Note that $\epsilon_{ij}^{0} = \sqrt{\epsilon_{i}^{0}\epsilon_{j}^{0}}$ and $r_{ij}^{0} = \frac{r_{i}^{0}+r_{j}^{0}}{2}$, and they represent, respectively, the well depth and equilibrium distance for the pairs of ith side chain and j^{th} membrane grid atom.

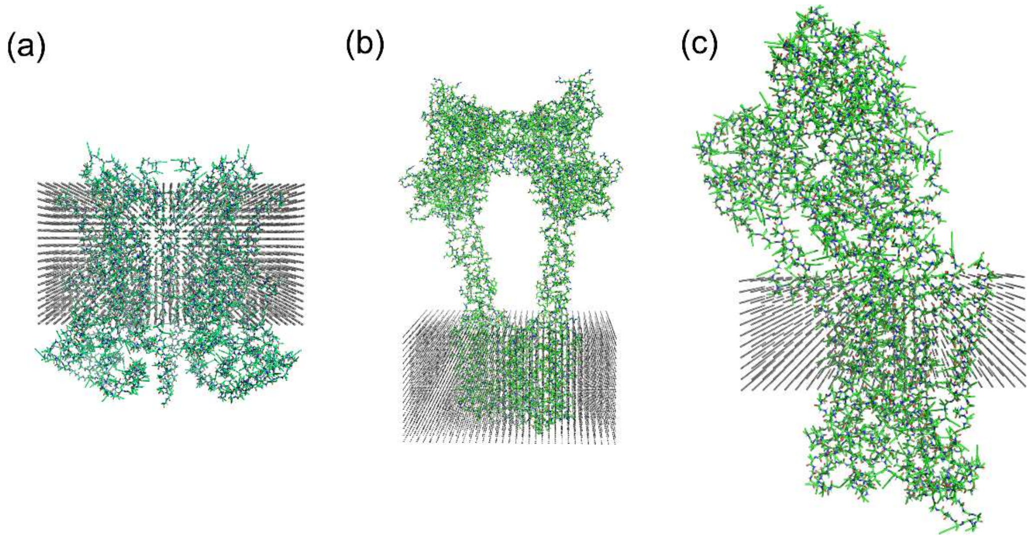

Figure S1. Representation of the structures sampled from the CG model with membrane environment. The membrane particles are colored grey. (a) The inactive state of TMEM16A channel. (b) The inactive state of mGlu2. (c) E2P state of P4-ATPase.

Table S1. The free energy of mutations with inactive state and active state (kcal/mol).

	$G_{inactive}$	G_{active}	$G_{inactive}$-G_{active}
I550A	-203.35	-208.47	5.12
I551A	-208.78	-194.09	-14.69
K588S	-203.10	-207.20	4.10
I641A	-203.63	-207.65	4.02
K645S	-207.81	-212.19	4.38
Q649A	-211.15	-212.98	1.83
WT	-200.74	-203.95	3.21
